# Selective Diversity in RNA Viruses: Do They Know How to Evolve? A Hypothesis

**DOI:** 10.1002/bies.202400281

**Published:** 2025-02-07

**Authors:** Lev G. Nemchinov

**Affiliations:** ^1^ Molecular Plant Pathology Laboratory U.S. Department of Agriculture, Beltsville Agricultural Research Center Beltsville Maryland USA

**Keywords:** genetic variations, mutation, RNA viruses, recombination, selective diversity

## Abstract

Genetic diversity of viral populations is almost unanimously attributed to the build‐up of random mutations along with accidental recombination events. This passive role of viruses in the selection of viable genotypes is widely acknowledged. According to the hypothesis presented here, populations of steady‐state error copies of a master viral sequence would have a dominant mutant rather than a random pool of heterogeneous viral genomes with changes scattered uniformly without any preferential distribution. It would let viruses face the selection stage of host surveillance having a preceding set of potential survivors or “guard” genomes among an ordinary cloud of random quasispecies.

## Graphical abstract

Among many sophisticated things viruses “learned” during their evolution, could be a simple mechanism for preferential accumulation of genetic variability in regions beneficial for virus survival. Identification of the major players coordinating this selective diversity could find applications in the development of effective prophylactic and therapeutic measures against viral infections.

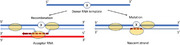

**Abbreviations**
HCV: hepatitis C virus
HIV: human immunodeficiency virus
HVR1: hypervariable region 1
RdRp: RNA‐dependent RNA polymerases
SARS‐CoV‐2: severe acute respiratory syndrome coronovirus

## 1 Introduction

Viruses are obligate parasites and cannot exist without the host. During their life cycle they are under continuous pressure of host surveillance prompting viruses to evolve toward escape mutants capable of counteracting host defenses. The invulnerability of some of the most threatening human RNA viruses, like severe acute respiratory syndrome coronovirus 2 (SARS‐CoV‐2), hepatitis C virus (HCV), human immunodeficiency virus (HIV), influenza viruses, and others depends to a great extent on their continuous genome diversity [1, 2, 3, 4]. In general, viral antigenic determinants that are responsible for immunologic escape are known or have been proposed. Finding an exact pattern of their heterogeneity would be a baseline contribution to the prophylaxis and treatment of these infections: intervention of high genetic variability in sequences encoding principal neutralizing epitopes either therapeutically or preventively could produce homogenous viral populations susceptible to the host immune surveillance or vaccination.

It is generally accepted that genetic complexity and diversification of RNA viruses depend mostly on the low level of fidelity of viral RNA‐dependent RNA polymerases (RdRp) and reverse transcriptases, their non‐efficient proofreading‐repair activities, and the recombination process between genetic variants [4, 5, 6, 7, 8]. The outcomes of error‐prone replication are mutations resulting from the incorporation of incorrect nucleotides, template misalignments, RdRp slippage, and so forth [5]. Recombination is an exchange of genetic material from different origins, which could be parental viral genomes and their derivatives, genomic fragments of viruses with segmented genomes (reassortment), non‐related viral genomes, and cellular RNA. It can occur via a copy‐choice (template switch) mechanism [6, 9, 10, 11] or be of non‐replicative nature. Recombination supplements the diversity of viral populations and is important for their evolutionary upgrades.

With rare exceptions, genetic diversity of viral populations is almost unanimously attributed to the build‐up of random mutations along with accidental recombination events [4, 5, 7, 8]. Thus, a conventional order of genome variability in RNA viruses appears to be the following: random genome variations→ quasispecies → survival of the fittest. Such a passive role of viruses in the selection of viable genotypes, that is, only generation of a randomized genetic drift, is widely acknowledged. Meantime, a random pool of heterogeneous viral RNAs would consecutively lead to the detection of quasispecies with changes scattered uniformly throughout the genome without any preferential accumulation in the regions beneficial for virus adaptability, which is not always the case [12, 13, 14]. For example, overall quasispecies variability of hepatitis C virus (HCV) is attributed mainly to the viral principal neutralization epitope, hypervariable region 1 (HVR1) of the envelope protein E2 [1, 14, 15]. HVR1 is a “hotspot” of sequence variations and comprises the most extreme degree of polymorphism and complexity in closely related but genetically non‐identical virions of HCV [1, 14, 15]. A question remains if this site‐specific complexity is present only in the HCV genomes that survived antibody‐driven selective pressure, or does it reflect uneven distribution of mutational events among the genomes of all viral quasispecies? If the latter is true, there could be a mechanism responsible for the variation of mutation and recombination rates along the viral genome.

## 2 Main Text

Keeping in mind many unique strategies that a number of viruses have developed to bypass host antiviral defenses, this assumption seems quite possible. In other words, a virus can play an active role in the distribution of variability in the favorable loci of its genome, thus self‐controlling a functional genetic diversity in the population rather than acting blindly to supply raw materials for adaptation [5]. This hypothesis implies a specific pattern by which viral replication machinery can be consistently affected at the same sites of the genome, defined during the course of virus evolution. As a result, “hotspots” of recombination and mutations would be positioned at the specific, virus‐directed locations vital for pathogen survival. Hence, the order random genome variations → quasispecies → positive selection would undergo a significant change: non‐random genome variations → quasispecies → positive selection. That is, virus survival would not entirely (passively) depend on the host‐driven selective pressure but would also be facilitated by the virus itself.

Under this scenario, even a population of steady‐state error copies of a master viral sequence [4] would have a dominant mutant with high adaptive capacity rather than a completely random pool of heterogeneous viral genomes with changes scattered uniformly without any preferential accumulation. It would let viruses face the selection stage of host surveillance already having a set of preceding potential survivors, “guard” genomes among an ordinary cloud of random quasispecies. The mechanism could appear in the early period of evolution of RNA viruses and be adopted for use by positive selection. If such is the case, it may open new perspectives for the exploration of genome adaptability and evolution of RNA viruses as well as for potential antiviral strategies. The existence of unique molecular machinery that regulates a preferable site‐directed heterogeneity in RNA viruses is as real as any other complex biochemical mechanisms developed by viruses to circumvent host defenses: viral security proteins, silencing suppressors, modulation of apoptosis, hijacking host defense proteins, and so forth [16, 17, 18, 19, 20, 21]. Indeed, “viruses have ‘studied’ immunology over millions of years of coevolution with their hosts” to develop their escape mechanisms [22].

According to this hypothesis, a precisely localized, virus‐controlled pausing of RdRp during RNA replication is a key factor in the subsequent non‐random distribution of genetic variability in the viral genome. A prediction that stopping of RdRp may initiate template switching during RNA recombination events is not novel [11, 23]. RdRp stalling has also been linked to the fidelity‐controlling mechanism possibly involved in optimization of virulence by modulating mutation rates [24]. RdRP pausing may be initiated by several tentative mechanisms, including specific RNA sequence motifs, or pausing signals [11], secondary structures, RdRp slippage followed by the incorporation of non‐templated nucleotides, and regulatory proteins [23, 24, 25, 26, 27]. Although being one of the potential causes, secondary structures may also restrict sequence variability and divergence of viral genomes [28]. Irrespective of what the exact reason for RdRp pausing is, this process gains new meaning if assumed that regions promoting it are not scattered spontaneously through viral genome but rather precisely positioned as being crucial for virus survival.

**FIGURE 1:**
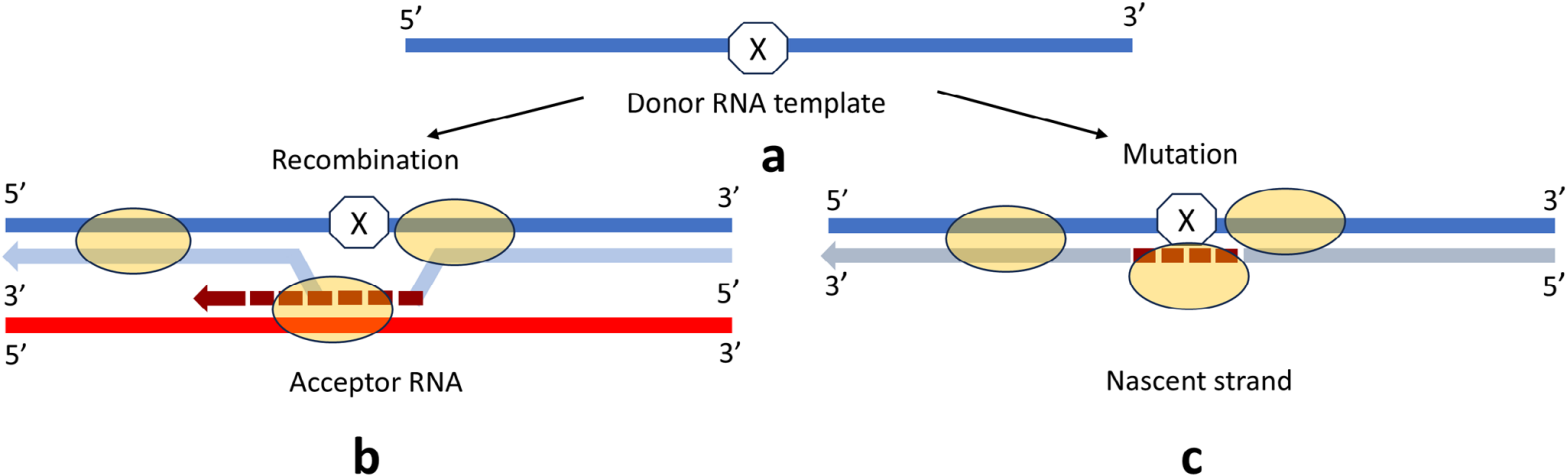
Simplistic representation of the tentative mechanism of selective diversity in RNA viruses. (a) Donor RNA template (positive– or negative‐sense viral RNA (+ssRNA shown) is inaccessible to RNA dependent RNA polymerase (yellow oval, RdRp) during the synthesis of a nascent strand (light blue line) because of the unknown RdRp pausing signal (octagon) located at the defined locus of the viral genome important for the generation of escape variants. Pausing of the RdRp can trigger induction of a copy‐choice mechanism of recombination (b), (dotted dark red line) or otherwise an imperfect fidelity of replication (c), (dotted dark red line) [22]. When the enzyme reaches a pausing signal, it switches from donor to acceptor (red line) template (recombination) or misincorporates random NTPs (mutation).

Pausing of the RdRp due to one of these mechanisms may initiate a copy‐choice mechanism of recombination (template switching) or otherwise imperfect fidelity of replication, that is, the insertion of non‐templated nucleotides in an unknown reaction that does not require base pairing to the template. While switching a template may occur by means of reassociation with the original template at a different position, or with homologous and non‐homologous templates thus generating RNA of mixed ancestry, [27] misincorporation could potentially result from a low‐fidelity pathway initiated by RdRp pausing [24]. After the hypothetical signal causing RdRp to pause is bypassed, the enzyme continues synthesis of a nascent strand using the respective RNA molecule as a template (Figure 1).

At first glance, this model looks similar to the described patterns of RNA variations [4, 5, 6, 7, 8, 9, 10, 11]: template‐switching and error‐prone replication apparently remain mechanisms of choice. The main difference is that the frequency and location of recombination events and base misincorporations in the growing nascent strand would rely on the specific features of donor RNA, such as a precise portion of the template that caused RdRp pausing and would not be promoted by any incidental sequence similarities or structural appearances of the randomly occurring acceptor strands [11]. Simply put, it would be a virus‐mediated intervention of the RNA elongation process catalyzed by RdRp followed by incorporation of non‐templated nucleotides or dissociation from the primary template. Speculatively, these unknown signal(s) triggering RdRp pausing could be selectively positioned by the virus to promote a high genetic variability in the regions of the genome critical for survival.

Thus, viruses would have a developed capacity to control rates of mutation and recombination events in the target area that would lead to their uneven distribution along the viral genome. In the end, the proportion of genetic variations will be much higher in a target segment that is responsible for viral persistence. It will indeed be a wide range of pre‐existing populations ready for environmental change and rapid selection [29], although these variants would already represent favorable mutants carrying heterogeneity in the vitally important loci of the genome as a result of non‐random mutation rates [30, 31].

In addition to plus‐sense single‐stranded (+) ssRNA, (−) ssRNA, and dsRNA viruses which replicate using RdRp, reverse‐transcribing ssRNA and possibly dsDNA reverse‐transcribing viruses could possess a comparable mechanism, wherein virus‐mediated genetic diversity would be dependent on the imprecision of viral reverse transcriptase. It is reasonable to assume that the proposed hypothesis could relate to DNA viruses as well, although lower genome variability, more accurate replication machinery, and sophisticated recombination process would imply different mechanisms [5].

## 3 Conclusions

Whereas the hypothesis presented here conflicts with the conventional evolutionary wisdom endorsing randomness of mutations in any genome, it does not neglect this fundamental postulate of biology but rather suggests that uneven distribution of mutation rates may lead to a more consistent emergence of adaptive features in the viral genome.

Notwithstanding, the directionless nature of mutations is being challenged by some of the past [32] and recent discoveries [30, 34] and opinions [35]. Likewise, the existence of a mutation bias guided by the molecular determinants of fidelity in the polymerase of RNA viruses and responsible for the generation of a favorable spectrum of viral sequences has been previously suggested [36].

If true, the proposed hypothesis of selective genomic diversity can represent a functionally new subtype of known genetic variations in RNA viruses and a mobile survival tool in their everyday battle against host immune surveillance leading to long‐range successful evolutionary upgrades. Indeed, “the evolutionary process may have been channeled, in nature as in the laboratory, toward repeated selection of the simplest solution to a biochemical problem” [37]. Identification of the major players coordinating selective heterogeneity in viral genomes could find many applications in the development of effective prophylactic and therapeutic measures against viral infections.

## Ethics Statement

No human participants were used in this study.

## Consent

The author consents to the publication of the manuscript.

## Conflicts of Interest

The author declares that he has no competing interests.

## Data Availability

No datasets were generated or analyzed during the current study.
